# Surgical and oncological results after rectal resections with or without previous treatment for prostate cancer

**DOI:** 10.3389/fsurg.2024.1298865

**Published:** 2024-02-01

**Authors:** T. Tomminen, H. Huhtala, S. Kotaluoto, T. Veitonmäki, E.-V. Wirta, M. Hyöty

**Affiliations:** ^1^ Department of Gastroenterology and Alimentary Tract Surgery, Tampere University Hospital, Tampere, Finland; ^2^ Faculty of Social Sciences, Tampere University, Tampere, Finland; ^3^ Department of Urology, Tampere University Hospital, Tampere, Finland

**Keywords:** rectal cancer, prostate cancer, rectal resection, oncological results, permanent stoma

## Abstract

**Introduction:**

Previous treatment for prostate cancer (PC) may potentially affect the surgical and oncological outcomes of subsequent rectal cancer surgery, but there are only a few studies regarding this particular group. In this study, we present the 3-year surgical and oncological results of rectal cancer patients who had received previous treatment for PC at a single Finnish tertiary referral centre.

**Material and methods:**

Data regarding all male patients diagnosed with rectal cancer and treated at Tampere University Hospital (TAUH) between 1997 and 2016 were gathered from medical records. In total, this study included 553 rectal cancer patients who underwent curative surgery, and 54 of them (9.8%) had a prior history of treatment for prostate cancer.

**Results:**

Patients in the PC group were older and had more comorbidities compared with those in the non-PC group. The PC patients had a significantly higher risk of permanent stoma compared with the non-PC patients (61.5% vs. 45.2%, respectively, *p* = 0.025). The PC patients seemed to have lower tumours than the non-PC patients (87% vs. 75%, respectively, *p* = 0.05). Overall, the 3-year overall survival (OS) for the PC and non-PC patients was 74.1% and 80.6%, respectively. No significant differences were observed between the study groups even in the age-adjusted comparison [hazard ratio (HR): 1.07, confidence interval (CI) 95%: 0.60–1.89]. In the univariable analysis, radically operated patients without a history of PC exhibited an improved overall survival, (HR: 2.46, 95% CI: 1.34–4.53, *p* = 0.004). However, only a higher age-adjusted Charlson comorbidity index (CCI) and a low tumour location (<10 cm) were found to have an independent prognostic impact on worse OS in the multivariable analysis (HR: 1.57, 95% CI: 1.36–1.82, *p* < 0.001 and HR: 2.74, 95% CI: 1.32–5.70, *p* = 0.007, respectively). No significant differences were observed between the groups in terms of disease-free or local recurrence-free survival.

**Conclusion:**

Rectal cancer is more frequently found in the middle or lower part of the rectum in patients who have previously received treatment for prostate cancer. These patients also have a higher likelihood of requiring a permanent stoma. In radically operated rectal cancer, the PC group had a worse OS rate, according to the univariable analysis. However, the only independent prognostic factors for a worse OS that were highlighted in the multivariable analysis included a higher CCI and a low tumour location.

## Introduction

Prostate and rectal cancers are the two most common pelvic malignancies in men (1, 2). The curative treatment options for prostate cancer (PC) are limited to either prostatectomy or radiotherapy (3). However, patients who have received radiotherapy for pelvic cancer have an increased risk for rectal cancer (4–7). Studies have demonstrated that neoadjuvant radiotherapy decreases the risk of local recurrence in stage II and III mid and low rectal cancer (6). Following high-dose radiotherapy for prostate cancer, administering further pelvic radiation is typically impossible due to locoregional toxicity; thus, neoadjuvant therapy for rectal cancer must be omitted. Furthermore, these patients have been suggested to have increased surgical morbidity and generally impaired treatment outcomes compared with prostate cancer naive patients (8, 9). So far, only one large multicentre retrospective study has reported prostate cancer treatment to be independently associated with worse overall survival (OS), disease-free survival (DFS), and local recurrence-free survival (LRFS) (10).

Our aim is to compare the surgical and oncological results of rectal cancer surgery in patients previously treated for prostate cancer compared with prostate naive patients.

## Patients and methods

**Figure 1:**
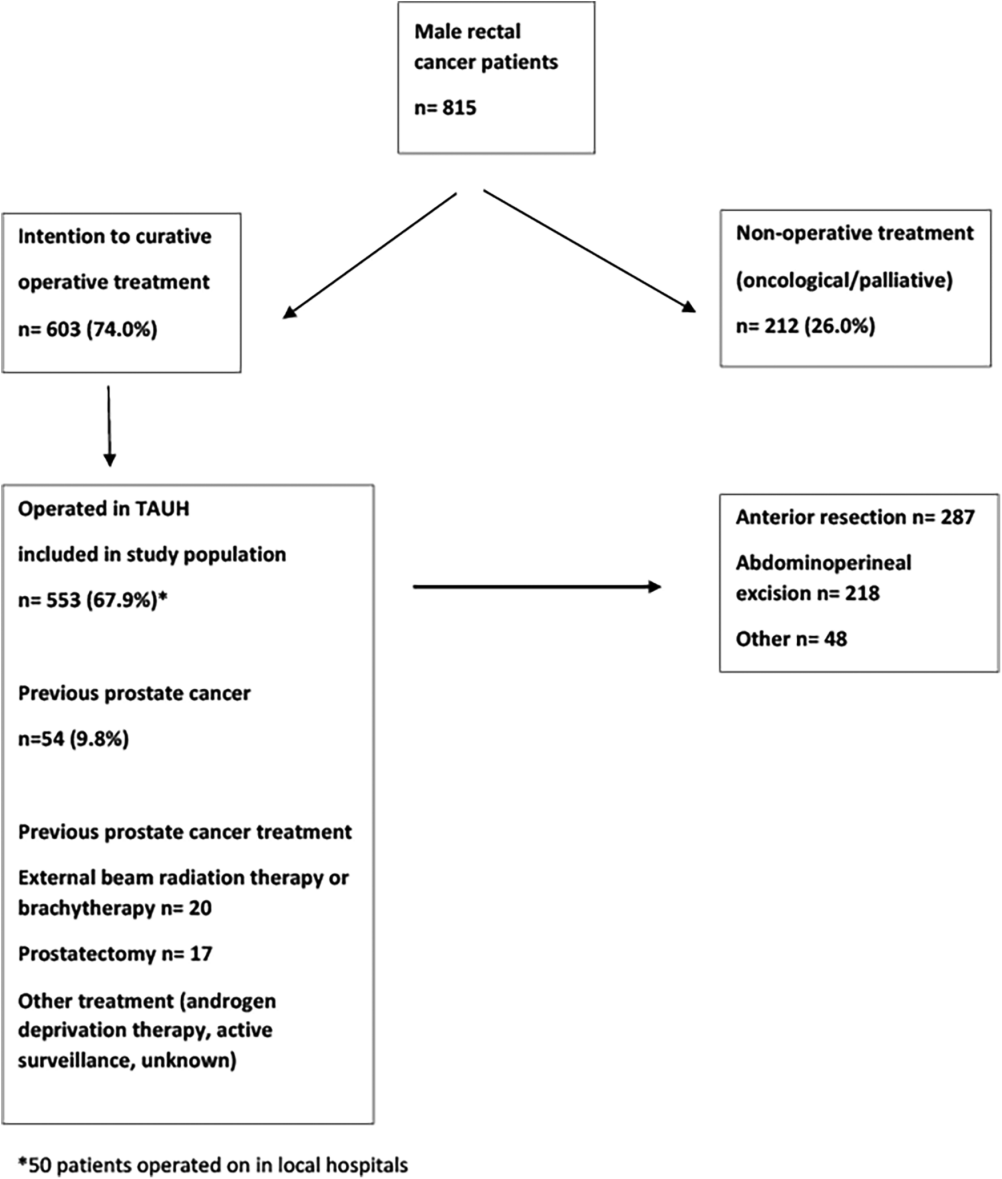
Flow chart of male rectal cancer patients treated at Tampere University Hospital (TAUH) between 1997 and 2016.

All male patients treated for rectal cancer (located 0–15 cm from the anal verge) at Tampere University Hospital were identified from medical records according to the ICD-10 classification. Tampere University Hospital is a tertiary referral centre for rectal cancer and also performs long-term follow-up. A total of 815 male patients were treated for rectal cancer in our institute between January 1997 and December 2016. The majority of the patients (603, 74.0%) underwent curative treatment, while 212 (26.0%) patients received only oncological or palliative treatment. A total of 50 patients were operated on in smaller units of the healthcare district and were not included in the analysis. The final study population consisted of 553 patients diagnosed with rectal cancer. Of these, 54 (9.8%) patients had a history of previously treated prostate cancer. Radiotherapy was used to treat prostate cancer in 20 (37%) patients, whereas radical prostatectomy was performed on 17 (32%) patients. A flow chart of patient selection is shown in Figure 1.

Clinicopathological data gathered from the medical records included age, BMI, comorbidities (CCI), neoadjuvant treatment, preoperative tumour stage, height of tumour from the anal verge, the type of surgery (laparoscopic vs. open surgery) and operation technique (anterior resection or abdominoperineal resection) used, the amount of operative bleeding, operative time, conversion to open surgery, Clavien–Dindo classification of complications, length of stay in the hospital, permanent stoma, circumferential lateral margin (CRM) positivity (lateral margin ≤1 mm), the amount of lymph nodes, postoperative tumour stage, time for local recurrence or metastases, and time of death. In addition, information concerning the previous prostate cancer treatment included the type of treatment used (radical prostatectomy, radiotherapy, androgen deprivation therapy, or active surveillance), the radiotherapy dose and the last day of radiotherapy, primary Gleason score, and PSA.

The dates and causes of death were obtained from the national registry of death certificate in the Finnish population (11).

### Treatment and follow-up

Prostate cancer treatment included standard external beam radiotherapy (EBRT) (70–78 Gy), prostate brachytherapy, radical prostatectomy, androgen deprivation therapy, or active surveillance. From 1996 to 2006, the standard radiotherapy dose was 70 Gy, which was subsequently increased to 78 Gy. A radical prostatectomy was performed using an open or robotic technique. The decision on treatment was evaluated by multidisciplinary consultation teams when necessary.

Short- (5 days) or long-course (5 weeks) neoadjuvant radiotherapy for rectal cancer was designated according to the radiological staging of the tumour. In T4 tumours with involved mesorectal fascia, patients were treated with long-course chemoradiotherapy (50.4 Gy, with concomitant 5-fluorouracil or capecitabine).

Short-course radiotherapy was indicated for T3–T4 or N1–N2 tumours, if the mesorectal fascia was intact. The treatment consisted of 25 Gy delivered in 5 consecutive days. Patients who had previously received radiotherapy for prostate cancer did not receive neoadjuvant treatment for rectal cancer but were otherwise treated according to the same principles as patients without previous radiotherapy. Multidisciplinary teams held pre- and postoperative discussions for all patients. Patients meeting the criteria of being medically suitable and having lymph node involvement (N1–N2) or being in an advanced stage (T4 or with mesorectal involvement) were offered adjuvant chemotherapy for 3–6 months (CAPOX, single fluorouracil, or kabesitabin).

All patients were followed up according to hospital protocol for a duration of 5 years following operative treatment. Follow-up time was defined as the time interval from rectal cancer operation to death or to the end of follow-up (January 2019).

### Surgery

Patients were operated on either 10 days after the onset of the short-course (25 Gy) radiotherapy or 6–10 weeks after cessation of the long-course radiotherapy (50.4 Gy). Without neoadjuvant treatment, the operation was performed as soon as possible within 2–4 weeks.

Rectal resections [abdominoperineal excision (APR)] or anterior resections were performed through laparotomy or laparoscopic procedures. An APR was performed in cases where the tumour involved the sphincters or levator muscle, or rectal anastomosis was considered unsafe for the patient due to significant comorbidities. The amount of extended APR (ELAPE) could not be evaluated. Local transanal excision was considered sufficient in Tis or T1 tumours if the patient was unwilling or unfit for radical surgery. High rectal tumours (>10 cm) were subjected to partial mesorectal excision. Mid or low tumours (<10 cm) were treated according to the principles of total mesorectal excision (TME) (12). A temporary loop ileostomy or transverse colostomy was usually used in mid or low (<10 cm) anastomosis. The specimen was extracted by laparoscopic operations through a small Pfannenstiel incision. A presacral passive drain (Penrose) was used in mid or low anastomosis (<10 cm).

### Endpoints

The primary endpoints were the short-term overall survival, disease-free survival, and local recurrence-free survival within a 3-year period. OS refers to the interval between a rectal operation and death. DFS refers to the time between a surgical operation and the detection of metastases or local recurrence. LRFS is the time from the rectal operation to the recurrence of the disease in the anastomosis, pelvis, or perineum.

The secondary outcomes were major surgical complications, operative blood loss, operation time, anastomotic leakage, permanent stoma, and circumferential resection marginal (CRM) positivity. The Clavien–Dindo classification was used to categorize surgical complications into two groups: minor complications (CD: 1–2) and major complications (CD: 3–4) (13). According to the definition proposed by the International Study Group of Rectal Cancer, the anastomosis leakage was evaluated using grades A–C depending on the consequences and treatment of the leakage (14).

### Statistical analysis

The data are presented as medians and quartiles (Q_1_–Q_3_) for numerical variables and as numbers and percentages (%) for categorical variables and compared using *Χ*^2^ or Fisher exact tests. A two-sided *p*-value below 0.05 was considered statistically significant. The overall survival rate of the PC and non-PC patients was compared using Kaplan–Meier analysis with the log-rank test. Univariate and multivariable Cox regression analysis was used to calculate the hazard ratios (HR) with the 95% confidence intervals (95% CI) to identify factors associated with mortality and cancer recurrence. The statistical analyses were conducted with the SPSS 27 software (IBM Corp., Armonk, NY, USA).

## Results

The patients in the PC group were significantly older (75 vs. 68 years, *p* < 0.001) than the patients without prostate cancer treatment. In addition, the patients in the PC group had more comorbidities (CCI score 3–6, 20.4% vs. 6.0%, *p* < 0.001), also in an age-adjusted comparison. Clinical mesorectal fascia involvement or clinical lymph node stage could not be evaluated due to the predominant use of endorectal ultrasound for preoperative staging during the study period. The demographic and clinical characteristics are shown in Table 1.

**Table 1:** Demographics and clinical characteristics of operated rectal cancer patients with or without previous prostate cancer.

	Prostate cancer *n* = 54		No prostate cancer *n* = 499		*p*-value
	*n*/median	%/Q_1_–Q_3_	*n*/median	%/Q_1_–Q_3_	
Age (years)	74.5	67–81	68.1	62–76	<0.001
BMI	25.7	23–28	25.8	24–29	0.617
ASA					0.077
1–2	17	32.1	217	44.7	
3–4	36	67.9	268	55.3	
CCI score					<0.001
1–2	24	44.4	171	34.2	
3–6	11	20.4	30	6.0	
Age-adjusted CCI	5.50	4–6	4.00	4–6	
Primary PSA	10.0	6–17			
Gleason score 6 or under	11	26.8			
7	21	51.2			
8–10	9	21.9			
Treatment for prostate cancer					
Radiotherapy (brachytherapy 3)	20	37.0			
Prostatectomy	17	31.5			
Androgen deprivation therapy	7	13.0			
Active surveillance	1	1.9			
Unknown	9	16.6			
Prostate cancer radiotherapy dose (Gy)	70.0	66–70			
Delay from EBRT to operation (mean), month	58.1	29–65			
Rectal cancer neoadjuvant radiotherapy	20	37.7	263	53.8	0.026
Rectal cancer preoperative stage					
1–2	38	76.0	321	70.5	0.420
3–4	12	24.0	134	29.5	
Location of rectal cancer					
High (10–15 cm from AV)	7	13.0	124	24.9	
Mid to low (<10 cm)	47	87.0	374	75.0	0.05

The operative and surgical postoperative results are shown in Table 2. Hartmann's operation was performed for three (5.5%) patients in the PC group and for 24 (4.8%) patients in the non-PC group as a primary operation or after complications. In the primary rectal resection, 44.9% (217/483) of the non-PC group had a permanent stoma compared with 57.1% (28/49) of the PC group. In the non-PC group, 24 ileostomies remained unclosed, while only three ileostomies in the PC group were deemed permanent. Eventually, 61.5% of the PC patients and 45.2% of the non-PC patients ultimately required a permanent stoma (*p* = 0.025). The greater risk of permanent stoma in PC patients can be attributed to the location of the tumour. Specifically, 87% of the PC patients and 75% of the non-PC patients had low tumours (<10 cm), *p* < 0.001.

**Table 2:** Surgical morbidity of operated rectal cancer patients with or without previous prostate cancer at Tampere University Hospital between 1997 and 2017.

	Prostate cancer		No prostate cancer		*p*-value
	*N* = 54		*N* = 499		
	*n*/median	%/range	*n*/median	%/range	
Type of rectal cancer surgery					0.072
Anterior resection	21	38.9	266	53.3	
Abdominoperineal resection	25	46.3	193	38.7	
Hartmann's operation	3	5.5	24	4.8	
Proctocolectomy	2	3.7	7	1.4	
Transanal excision	3	5.5	9	2.2	
Type of surgery					0.158
Open	42	77.8	398	80.2	
Laparoscopic	12	22.2	96	19.4	
Conversion to open surgery^b^	1	1.9	31	6.2	
Intraoperative blood loss	400	200–750	350	150–500	0.072
Operative time	145	120–201	155	120–201	0.585
Length of stay in hospital (day)	7	6–9	8	7–9	0.286
Complications Clavien–Dindo III–V	7	13.0	78	15.7	0.602
30 day mortality	1	0.2	9	1.8	1.000
Anastomotic leakage^a^	1	4.8	20	7.5	0.621
Grade 3^c^	1	4.8	15	5.6	1.000
Permanent stoma	32	61.5	217	45.2	0.025
CRM pos, <1 mm	9	17.6 9/51	56	11.5 56/488	0.461
Number of lymph nodes	15	11–24	16	10–21	0.535
Postoperative stage					0.869
Stage 2	26	50.0	238	48.7	
Stage 3	21	40.4	197	40.3	
Rectal cancer differentiation Grade 2	39	73.6	354	73.0	0.984
Adjuvant treatment	21	39.6	225	46.0	0.375

^a^ Proportion of anterior resections.

^b^ Proportion of all operations.

^c^ WHO 2010 grading.

The CRM was positive in 11.5% (56/488) of the PC group and in 17.6% (9/51) of the non-PC group.

Major complications (CD III–V) occurred in 12.9% of the PC patients and in 15.7% of the non-PC patients. The anastomosis leakage rate was 4.8% for the PC patients and 7.5% for the non-PC patients (*p* = 0.602). Complications are presented in Table 3.

**Table 3:** Surgically treated patients (553) at Tampere University Hospital between 1997 and 2016 for rectal cancer; most severe complications, Clavien–Dindo III–V.

Complication	*N*	Prostate cancer *n* = 54		No prostate cancer *n* = 499		*p*-value
	All	*n*	%	*n*	%	
Number of patients		7	12.9	78/498	15.7	0.602
Leakage^a^	21	1/20	4.8	20/266	7.5	
Wound rupture	12	3	5.7	9	1.8	
Pulmonary, thromboembolic	11	0	0	11	2.2	
Ileus	9	0	0	9	1.8	
Postoperative bleeding, hematoma	7	2	3.8	5	1.0	
Stoma-related complications	5	0	0	5	1.0	
Urinary tract complications	4	0	0	4	0.8	
Missing data	6			6		
Death	10	1	1	9		

^a^ Proportion of anterior resections.

### Univariable survival analysis

The 3-year overall survival rate after a rectal operation was 73.6%. The hazard ratio (HR) for mortality in the PC group was 1.34 compared with the no-PC group (95% CI: 0.95–1.96). No significant differences were observed in the OS rate between the PC and non-PC groups (74.1% vs. 80.6%), not even in the age-adjusted comparison [HR: 1.069 (0.60–1.89)]. There were also no significant differences observed in DFS between the PC and non-PC groups (73.6% vs. 76.3%, *p* = 0.647). The median time of occurrence of metastasis or local recurrence after rectal operation was 16.3 months for the PC group (Q_1_–Q_3_: 8.02–29.0) and 12.4 months for the non-PC group (Q_1_–Q_3_: 7.98–26.0). Local recurrence was detected in 7.5% of the PC group and 3.4% of the non-PC group (*p* = 0.137). The median time for local recurrence was 22.0 months for the non-PC patients (Q_1_–Q_3_: 10.9–45.0) and 18.8 months for the PC patients (Q_1_–Q_3_: 12.5–24.4). There was no difference observed in the 3-year LRFS between the study groups (96.6% for the PC group vs. 92.5% for the non-PC group, *p* = 0.172). Three (3/20, 15.0%) of the PC patients with previous radiotherapy had local recurrence, whereas one of the non-irradiated PC patients had recurrence (1/24, 4.2%), *p* = 0.138.

**Figure 2:**
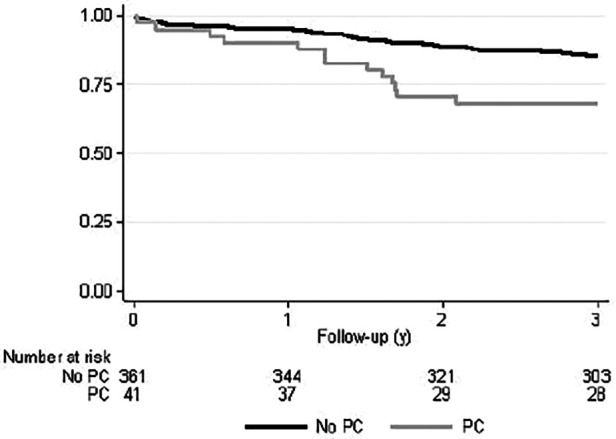
Kaplan–Meyer overall survival curve of radically operated rectal cancer patients with or without previous prostate cancer. *p* = 0.003.

We also analyzed the survival rates of radically operated patients as a subgroup analysis. Operation was determined radical if CRM was negative, the disease was not metastasized, and transanal operations were excluded. For these radically operated patients in the Kaplan–Meier survival analysis, the OS of the PC patients was significantly lower compared with the non-PC patients (*p* = 0.003) (Figure 2). However, no significant differences between groups in DFS or LRFS were observed. OS was also significantly lower in patients who had low (<10 cm) rectal tumours.

Patients with low tumours had more complications [CD: 1–2 108/302 (35.8%)] vs. high tumours [23/99 (23.2%)], *p* = 0.036. No significant difference in major complications was observed between the study groups: low tumours (45/302, 14.9%) vs. high tumours (13/99, 13.1%). Low rectal tumours seemed to have more poorly differentiated (Grade 3) cancers than high tumours [49/293 (16.7%) vs. 6/99 (6.1%), *p* = 0.27].

### Multivariable survival analysis

The Cox proportional hazards models for OS, DFS, and LRFS in all patients are shown in Table 4 and in radically operated patients in Table 5. The selected variables were considered to be the most important factors impacting the surgical outcome. The variables included in the multivariable analysis with an age-adjusted CCI were the history of prostate cancer, BMI, TNM stage, and location of rectal cancer, with the corresponding reference categories being no history of PC, BMI of 20–25, TNM stage I–II, and high location of the rectal cancer. A higher age-adjusted CCI was a risk factor for worse OS, with an HR of 1.57 (95% CI: 1.36–1.82), *p* < 0.001. Also, a low location of the tumour was an independent prognostic factor for a worse overall outcome, with an HR of 2.74 (95% CI: 1.32–5.70), *p* = 0.007. For disease-free survival, the TNM stage was the only independent prognostic factor, with an HR of 4.12 (95% CI: 2.46–6.90) for TNM stage III compared with TNM stage I–II, *p* < 0.001. Local recurrence was observed in only eight radically operated patients (2%), and therefore, the multivariable analysis of the LRFS was considered uninformative. A similar comparison of variables was used in a previous French study conducted by the GRECCAR group (10).

**Table 4:** Patient characteristics affecting overall, disease-free, and local recurrence-free survival in the multivariable analysis.

	No. of patients	No. of events	HR	95% CI	*p*-value
Overall survival (death events)					
History of prostate cancer					0.626
No	493	35	1		
Yes	53	18	1.36	0.82–2.21	
CCI (age-adjusted)	510	124	1.30	1.17–1.45	<0.001
BMI					0.212
20–25	206	63	1		
<20	25	9	1.50	0.74–3.01	
>25	310	72	0.84	0.45–1.08	
Postoperative stage					0.007
I–II	293	57	1		
III	217	67	1.63	1.15–2.33	
Location of rectal cancer					0.104
High (10–15 cm)	129	104	1		
Low (<10 cm)	417	119	0.70	0.50–1.08	
Disease-free survival					
History of prostate cancer					0.647
No	493	118	1		
Yes	53	14	1.14	0.65–2.01	
CCI (age-adjusted)	510	119	0.99	0.88–1.11	<0.001
BMI					
20–25	211	55	1		
<20	25	5	0.77	0.30–1.92	
>25	310	71	0.88	0.60–1.07	
Postoperative stage					<0.001
I–II	293	39	1		
III	217	80	3.20	2.18–4.70	
Location of rectal cancer					0.095
High (10–15 cm)	129	24	1		
Low (<10 cm)	417	107	0.68	0.441.07	
Local recurrence-free survival (local received)					
History of prostate cancer					0.172
No	484	17	1		
Yes	52	4	2.18	0.71–6.64	
CCI (age-adjusted)	510	19	1.13	0.85–1.5	0.41
BMI					0.44
20–25	206	7	1		
<20	24	2	2.81	0.57–13.8	
>20	306	12	1.24	0.96–1.04	
Postoperative stage					0.16
I–II	293	8	1		
III	217	11	1.92	0.77–4.80	
Location of rectal cancer					0.14
High (10–15 cm)	129	2	1		
Low (<10 cm)	417	19	0.33	0.77–1.45	

**Table 5:** Radically operated patient characteristics affecting overall and disease-free survival in the uni- and multivariable analyses.

	No. of patients	No. of events	Univariate		*p*-value	Multivariable		*p*-value
			HR	95% CI		HR	95% CI	
Overall survival (death events)								
History of prostate cancer								
No	358	52	1			1		
Yes	41	13	2.46	1.34–4.53	0.004	1.26	0.66–2.40	0.48
CCI (age-adjusted)	399		1.54	1.36–1.79	<0.001	1.57	1.36–1.82	<0.001
BMI								
20–25	150	21	1			1		
<20	18	4	1.77	0.61–5.15	0.30	1.72	0.59–5.03	0.32
>5	231	40	1.25	0.74–2.11	0.45	1.31	0.76–2.25	0.31
Postoperative stage								
I–II	248	37	1			1		
III	151	28	1.28	0.78–2.08	0.33	1.17	0.71–1.92	0.54
Location of rectal cancer								
High (10–15 cm)	98	9	1			1		
Low (<10 cm)	301	56	2.15	1.06–4.35	0.03	2.74	1.32–5.70	0.007
Disease-free survival								
History of prostate cancer								
No	358	62	1			1		
Yes	41	7	1.14	0.52–2.50	0.77	0.92	0.41–2.08	0.85
CCI (age-adjusted)	396		1.06	0.91–1.23	0.52	1.07	0.92–1.24	0.37
BMI								
20–25	150	28	1			1		
<20	18	3	0.97	0.30–3.20	0.97	0.98	0.30–3.24	0.98
>25	231	38	0.88	0.54–1.44	0.61	0.94	0.57–1.53	0.79
Postoperative stage								
I–II	248	21	1			1		
III	151	48	4.20	2.51–7-01	<0.001	4.12	2.46–6.90	<0.001
Location of rectal cancer								
High (10–15 cm)	98	12	1			1		
Low (<10 cm)	301	57	1.72	0.92–3.20	0.09	1.63	0.87–3.06	0.13

## Discussion

Our study aimed to clarify the impact of previous prostate cancer treatment on the surgical and oncological outcomes during the 3-year timeline following rectal cancer surgery. Rectal cancer patients who have previously been treated for prostate cancer are a special and demanding group. The male pelvis is narrower in structure, and the difference in distribution of intra-abdominal fat causes technical challenges compared with the female pelvic operations. Both radiotherapy for prostate cancer and radical prostatectomy cause problems for subsequent rectal surgery (8, 10). Fibrosis, fragility of tissues, and additional bleeding complicate the operation and increase the risk for postoperative complications. Laparoscopic rectal resections just started in our hospital during this study period. In the beginning, we had a very low threshold to perform open surgery, although the conversion rate was acceptable, which was 22.9%. This rate is in accordance with previous reports from the initial days of laparoscopic rectal resections (15, 16). There was a tendency to avoid anastomosis by doing abdominoperineal excision more freely in the PC group, although this difference was not statistically significant. One explanation could be the larger number of lower tumours in the PC patients. There was also a tendency toward more blood loss in the PC group. On the other hand, no significant differences were observed in the operative time, length of hospital stay, or complications. This can also reflect the right decision to perform open surgery and reduce anastomosis in patients with higher requirements.

Patients with prostate cancer were significantly older than patients without prostate cancer. A possible explanation for this is the natural course of these tumours. On average, prostate cancer is diagnosed in older patients (approximately 70 years) compared with rectal cancer (over 60 years) (2, 17). In addition, radiotherapy itself can induce secondary rectal cancer (4–7). Rombouts et al. (5) published in their population-based study from the Netherlands a median interval of 6 years from pelvic radiotherapy to rectal cancer. In our study, the mean interval to secondary rectal cancer after PC radiotherapy was 4.8 years.

Patients with former prostate cancer treatment had significantly more comorbidities than the non-PC patients. This difference was also shown in the age-adjusted comparison and is one possible reason for the worse outcome of these patients.

The treatment of prostate cancer had been radiotherapy for 42% of the patients, and 32% underwent radical prostatectomy. Because of previous irradiation, additional preoperative radiotherapy later for rectal cancer is hampered in a considerable portion of PC patients. Therefore, only 38% of the PC patients (compared with 54% of the non-PC patients) received preoperative radiotherapy for rectal cancer in this study. As preoperative radiotherapy improves the oncological results of rectal cancer, the inadequate preoperative treatment may lead to worse outcomes for patients with a history of prostate cancer (6, 18).

If preoperative radiotherapy has been omitted, there is some evidence that patients could, instead of radiotherapy, have only preoperative chemotherapy. Deng et al. (19) presented a study where a 3-year DFS following chemotherapy did not differ from neoadjuvant chemoradiotherapy. In our study population, neoadjuvant chemotherapy alone has not been used.

Lakkis et al. (10) showed in their study a significantly increased local recurrence rate in the PC group and a decrease in DFS and OS. Although the patients in the PC group were older, had more comorbidities, and had less preoperative radiotherapy for rectal cancer, no significant difference was found in the local recurrence rate in this study. The local recurrence rates for all patients (7.5% in the PC group vs. 3.5% in the non-PC group) and for radically operated patients (2.4% in the PC group vs. 2.0% in the non-PC group) were in accordance with previous literature (10, 20). In the univariable analysis, the overall survival rate was worse for radically operated PC patients, but the difference was not observed in the multivariable analysis.

The surgery for colorectal cancer is associated with a high risk of postoperative infectious complications, particularly surgical site infections (SSIs). SSIs are associated with longer hospital stays, negative economic impact, readmission, increased morbidity, sepsis, and even death (21). Anastomotic leakage is the most frequent cause of serious septic complications (22). Anastomotic leakage deteriorates the surgical result after rectal resection by increasing fibrosis in the anastomotic region (23–25). Leakage is also associated with a worse oncological outcome (20). Our study assessed the extent of leakage based on the grading system proposed by Rahbar et al. (14), considering also clinically asymptomatic patients if there was any doubt about the presence of leakage. The leakage rate in our study was relatively low, with the PC and non-PC patients experiencing a leakage rate of 4.8% and 7.5%, respectively. This may be attributed, in part, to our strict perioperative selection process to avoid primary anastomosis in high-risk patients and prevent the occurrence of adverse events. This is indicative of the limited occurrence of anterior resections with primary anastomosis in our study (39% for PC and 53% for non-PC patients). Contrary to a recent French multicentre study (10), the differences in leakage rate between the study groups were not found in our study (10). The reason for this may be attributed to the lower numbers and limited occurrence of primary anastomosis in our study. In a recent national cancer registry analysis from Sweden, the leakage rate for rectal resections in patients who were previously irradiated for prostate cancer was 20%. However, their patients exhibited rather good health with early cancer stages (ASA 1–2, 71% and Stage 1–2, 61%) (26).

One potential approach to reduce the morbidity associated with low colorectal anastomosis is to perform a delayed coloanal anastomosis. Recent evidence suggests that the delayed coloanal anastomosis technique is feasible and is associated with a low rate of complications (27, 28).

Appropriate TME quality is crucial for good operative results in rectal resections (29, 30). CRM positivity is a strong predictive parameter. In the non-PC group, the resection margin was clean (R0) in 88.5% of the cases, while in the PC group, it was clean in 82.4% of the cases. There were no significant differences between the study groups. These numbers are in accordance with the literature, considering that all patients were males (10, 31–33).

There was no difference in local tumour spread between the study groups, since 40.4% of the PC group and 40.3% of the non-PC group had stage III tumours, and the use of adjuvant treatments was similar.

There were no significant differences in the rate of abdominoperineal resections between the study groups. Nevertheless, after the long-term follow-up, the PC patients were shown to have a significantly higher incidence of permanent stomas. This is in concurrence with an earlier population-based study conducted by Feinberg et al. (34) in Canada. In our study, the PC and non-PC patients had no differences in complications following the primary operation. However, the PC patients were older and had more comorbidities, which may account for the higher frequency of planned temporary stomas transitioning into permanent ones.

For radically operated patients (transanal procedures, CRM positivity, and metastasized diseases excluded), a previous history of PC was associated with a worse OS. This is probably related to the higher age and associated increased comorbidity of the PC patients. The multivariable analysis revealed that the low location of the tumour was an independent risk factor for a worse OS but not for DFS. Although there was a slight difference in complications and tumour grade, the difference in survival could not be explained.

Our study has several strengths. The hospital where the study took place is a specialized centre that receives a large number of patients referred specifically for rectal issues. The patients included in the study were gathered consecutively without selection. All the patients underwent surgery and were thereafter monitored at the same hospital, and the gathered data can be considered as real-life outcomes.

The basic operative TME technique has been used constantly throughout the study period. Preoperative cancer staging was done by endoultrasound in the early years and for the last 10 years with MRI-staging according to standard diagnostic methods. Neoadjuvant radiotherapy has been routinely used all over the study period based on the study conducted by Påhlman et al. (18, 35), and postoperative adjuvant treatment was administered according to ESMO guidelines (36).

This study also has some limitations. The study was retrospective, and the number of rectal cancer patients with previous treatment for prostate cancer was relatively small. Because of the small number of prostate cancer patients causing possible type 1 errors, the results should be verified in a larger study. The long study period of 20 years can introduce bias as a result of changes and improvements in multimodal cancer treatment. The basic operative treatment for rectal cancer remained consistent throughout the study period, following the TME principles.

In conclusion, rectal cancer in patients with a history of prostate cancer was more frequently localized in the mid or lower rectum, with limited possibilities for standard neoadjuvant treatments. In radically operated rectal cancer, the PC group had a worse OS, according to the Kaplan–Meier analysis. However, in the multivariable analysis, only the location in the lower rectum and higher CCI were the independent risk factors for a worse OS. Patients with previous treatment for prostate cancer were older, with more comorbidities, and had an increased risk for permanent stoma. This should be considered when planning the operation strategy.

## Data Availability

The raw data supporting the conclusions of this article will be made available by the authors, without undue reservation.
